# Supplementary material for: Staff’s Knowledge and Self-Confidence in Difficult Communication: Evaluation of a Short Experiential-Based Training Program
Source: Nurs Rep. 2025 Feb 8;15(2):60. doi: 10.3390/nursrep15020060 (PMC11858279; doi:10.3390/nursrep15020060)
Supplement: Supplementary file 1 [file nursrep-15-00060-s001.zip › nursrep-3311214-supplementary.pdf]

Table S1. Thematic areas of the knowledge test.

Thematic areas	Statements (T/F)	Correct answers Pre intervention N=28 n (%)	Correct answers Post intervention N=30 n (%)
Setting up and conducting family meetings	It is important to explore what people know about their situation before providing information (T)	26 (92.8)	29 (96.7)
	Summarizing the content of the conversation helps to highlight and clarify the points discussed (T)	28 (100)	29 (96.7)
	It is not necessary to know the person's medical history if communication protocols (e.g., SPIKES, REMAP, PREPARED, etc.) are well mastered (F)	22 (78.6)	28 (93.3)
	During a conversation, professionals should regularly check their interlocutor's understanding (T)	28 (100)	29 (96.6)
	Ensuring privacy is not essential if a simple and clear language is used (F)	23 (82.1)	23 (76.7)
	In remote communication, the camera should frame the face and the upper third of the body (V)	23 (82.1)	26 (86.7)
	Planning a follow-up meeting allows the patient/family to reflect on what they would like to discuss and to promote aware treatment choices (T)	28 (100)	30 (100)
	The Family Conference usually involves only the doctor and the patient (F)	23 (82.1)	30 (100)
	In remote communication, professionals should be in a private environment while the context of their interlocutor is less relevant (F)	16 (57.1)	23 (76.7)
Communication barriers and effective communication strategies and techniques	All recommendations agree on personalizing communication (T)	27 (96.4)	30 (100)
	Communication skills can be acquired as well as technical skills (T)	15 (53.6)	24 (80.0)
	Technical language provides useful details to patient/family to fully understand the illness (F)	21 (75.0)	27 (90.0)
	The "I would like" technique is useful for communicating deterioration (T)	14 (50.0)	16 (53.3)
	It is important to fill silence promptly to avoid "dead times" (F)	16 (57.1)	17 (56.7)
	Bad news should be anticipated by a warning signal to give the person enough time to get prepared (T)	19 (67.8)	26 (86.7)
	Pointing out what's wrong with your interlocutor is the starting point for effectively deescalating conflicts (F)	12 (42.8)	14 (46.7)
	A direct communication style with no gradualness in providing information is recommended to avoid misunderstandings and false hopes (F)	15 (53.6)	26 (86.7)
	It is necessary to avoid defeatist or overly pessimistic statements such as "there's nothing more we can do" (T)	22 (78.6)	29 (96.7)
	The Family Conference includes a time of preparation to discuss the case with colleagues and establish the role of everyone in the meeting (T)	19 (67.8)	25 (83.3)
	The silence encourages reflection on the provided information when not excessively prolonged (T)	24 (85.7)	27 (90.0)
	It can be useful to present numbers or percentages when providing clinical information (F)	19 (67.8)	20 (66.7)
Accommodating emotions and care preferences of the resident and/or their family carers	Denial of disease progression is common in patients with neoplastic or chronic-degenerative conditions and their family carers (T)	25 (89.3)	23 (76.7)
	Managing denial and offering support improves patients' and family carers' understanding on the information provided (T)	23 (82.1)	24 (80.0)

The fear of not being able to accommodate the interlocutor's emotions is one of the major obstacles in difficult communication (T)	25 (89.0)	25 (83.0)
Patients and/or families tend to forget information they do not want to accept (T)	25 (89.3)	27 (90.0)
Encouraging positive expectations helps define care goals consciously (T)	5 (17.8)	9 (30.0)
Changes in face expression, tone of the voice, and attitude are among the main indicators of anger/aggressiveness (T)	24 (85.7)	30 (100)
Individual interviews are preferable to group interviews when family carers have conflicting care preferences (F)	6 (21.4)	13 (43.3)
A relationship of mutual trust is useful but not essential to explore personal care preferences (F)	11 (39.0)	16 (53.0)
It is not always possible to identify a solution to solve conflicts (F)	11 (39.3)	12 (40.0)

Acronym: F, false; T, true.

Note. The correct answer is reported in brackets.

Table S2. Satisfaction with the communication skills training program.

	Neutral N (%)	Agree N (%)	Strongly agree N (%)	Mean (SD)*
**Organization of the training**				**4.6 (0.6)**
Relevance of the educational material (n=30)	1 (3.3)	8 (26.7)	21 (70)	4.7 (0.5)
Adequacy of physical spaces and technical equipment (n=30)§	2 (6.7)	9 (30)	18 (60)	4.4 (0.9)
Training content in line with the program (n=30)	-	10 (33.3)	20 (66.7)	4.7 (0.5)
Teachers' ability to promote interest (n=30)	1 (3.3)	10 (33.3)	19 (63.3)	4.6 (0.6)
**Usefulness of training methods in improving communication skills**				**4.5 (0.6)**
Lectures (n=29)	1 (3.3)	15 (50)	13 (43.3)	4.4 (0.5)
Brainstorming sessionc(n=30)	3 (10)	10 (33.3)	17 (56.7)	4.5 (0.7)
Video-cases (n=30)	2 (6.7)	9 (30)	19 (63.3)	4.6 (0.6)
**Professional relevance**				**4.7 (0.5)**
Transferability of knowledge in daily practice (n=30)	1 (3.3)	7 (23.3)	22(73.3)	4.7 (0.5)
Daily practice improvement after the training (n=30)	-	10 (33.3)	20 (66.7)	4.7 (0.5)
**Overall satisfaction**				**4.6 (0.6)**

* Likert scale 1-5 (1=strongly disagree; 5=strongly agree)

§One participant strongly disagree.
